# Renal Cell Carcinoma Update: News from the AUA, EAU, and ASCO Annual Meetings 2011

**DOI:** 10.5402/2012/748235

**Published:** 2012-06-10

**Authors:** Andres Jan Schrader, Sandra Steffens

**Affiliations:** ^1^Department of Urology, Ulm University Medical Center, Prittwitzstrasse 43, 89075 Ulm, Germany; ^2^Department of Urology, Hannover University Medical School (MHH), 30625 Hanover, Germany

## Abstract

Renal cell carcinoma (RCC) is one of the most important urologic malignancies with a continuously growing incidence and health economic relevance. In 2011, several hundred articles, abstracts, and lectures have been presented at the leading global urooncologic congresses. This review was composed to give an overview on the flood of novel findings dealing with diagnostics and therapy of both localized and advanced RCC. The most clinically relevant data are discussed in detail.

## 1. Incidence and Prevalence

In 2011 renal cell carcinoma (RCC) still received a great deal of attention at the major urological and oncological congresses worldwide. Though RCC is only the third most common urologic malignancy after prostatic and urothelial cancer, it accounts for more than 50,000 new cases in the USA and approximately 15,000 in Germany each year [1]. It takes a high death toll because of its often aggressive growth pattern, high rate of early metastasis, and the lack of curative therapy in advanced stages: in Germany alone, about one third of all RCC patients (*n* = 5,101) succumbed to their disease in 2008 [2]. The incidence has increased significantly in the last 30 years.At the last congress of the American Urological Association (AUA), this significantly increased incidence was discussed by a St. Louis research group and published by Nepple and Strope [3]. It was evaluated in 63,843 RCC patients by a retrospective analysis of the SEER (*Surveillance Epidemiology and End Results*) database from 1975 to 2006. The authors showed that the incidence increased in the USA from 7.4 to 17.6 per 100,000 population between 1975 and 2006. The mean annual increase in incidence was 3.6% from 1976 to 1990. This trend declined slightly in the years that followed. Thus, the mean annual increase in incidence was only 2.9% from 1991 to 2006. Analysis of the SEER data also revealed that the increase in incidence mainly affected older patients up to 1990 and younger ones after 1991. This also meant that, at the time of diagnosis, the majority of RCC patients were over 65 until 1991, whereas 55.3% were under 65 in 2006. The authors could not determine whether this shift towards younger age at diagnosis can be attributed to earlier tumor detection or earlier tumor development [3].

## 2. Localized RCC

Most congress contributions dealing with localized RCC focused on the following questions.

- Which RCC patients can and should be managed with an active surveillance strategy?
- When can and should a renal tumor biopsy be performed?
- What are the advantages and disadvantages of radical versus partial nephrectomy?
- What is the optimal access and best surgical procedure for partial nephrectomy?
- Are there alternative local ablation techniques equivalent to surgery?
- What is the value of new predictive or prognostic markers for patients with localized RCC?

### 2.1. Active Surveillance

*Active surveillance *naturally offers a number of significant advantages over an active treatment procedure. The patient is spared a surgical trauma and the associated perioperative risk. Moreover, renal function does not deteriorate, at least not primarily, since there is no loss of functioning nephrons by surgery/ablation. On the other hand, there are also thought-provoking arguments against an *active surveillance* strategy. Living with a potentially malignant renal tumor causes many patients considerable psychic stress. Then there are also the costs of regular follow-up imaging and the associated radiation exposure (30–90 mSv per CT scan). Moreover, we still have no large prospective studies with the long-term results of a *surveillance* strategy in RCC, nor do we have markers that can adequately predict progression of a small localized kidney tumor under *active surveillance*. 

Tsivian et al. [4] from Durham presented a retrospective monocenter analysis of data they collected from 2000 to 2008 in 243 patients who had received a partial nephrectomy for a small solid renal tumor. Tumors had a mean size of 2.9 cm in this patient population and were ultimately malignant in 73.7% of the cases. Sixty percent of tumors <2 cm and 77.6% of those >2 cm were malignant in this cohort. The authors concluded that an *active surveillance* strategy can be justified particularly for patients with tumors <2 cm since 40% of even the solid renal tumors were benign in this patient population. However, in a considerably larger study published in the Journal of Urology in 2003, Frank et al. [5] reported that the percentage of malignant renal tumors increases significantly with the tumor size. Small solid tumors <1 cm were benign or malignant in approximately equal proportions, but only about 22% of renal tumors >1 cm were benign. 

Predictors of systemic metastasis of small solid renal tumors were investigated by the study group around Smaldone from Philadelphia [6]. At the AUA Annual Congress, they presented a meta-analysis of 6 studies comprising 880 patients with a follow-up of 15 to 48 months. The tumors had a mean initial size of 1.7 to 7.2 cm and an annual growth rate of 0.1 to 0.7 cm. This meta-analysis revealed a particularly high risk of systemic metastasis in patients with small solid tumors, who tended to be older, as well as in those whose tumors had a larger diameter or volume, or those with a higher annual growth rate (0.8 cm versus 0.3 cm). Smaldone et al. [6] concluded that younger patients with small tumors as well as patients whose tumors have a low growth rate or none at all were optimal candidates for a *surveillance *strategy. 

However, Nguyen and Gill [7] already demonstrated the danger of even small renal tumors in a publication based on SEER database data from 2009. An evaluation of 24,253 patients revealed that 1.4% of all solid tumors <1 cm and even 7.4% of all tumors with a diameter of 3-4 cm (formally still stage T1a tumors) had already metastasized at the time of diagnosis. The percentage increased with tumor size, ranging up to 22% for 6-7 cm tumors. 

Approaching the problem from a completely different angle, Chang et al. [8] examined predictors of non-RCC-related death in patients with localized RCC in order to identify good candidates for an *active surveillance *strategy. Their retrospective analysis of 1,101 patients with localized RCC demonstrated that, during a median follow-up of 62 months, non-tumor-related mortality was naturally higher among older patients (particularly those >70) as well as those in a reduced general condition with significant comorbidities (ASA score: 3/4 versus 1/2, hazard ratio (HR): 3.3; *P* = 0.003). These data seem realistic and reasonable. In contrast to Smaldone et al. [6], Chang et al. [8] come to the conclusion that older patients with significant comorbidities have a higher risk of non-tumor-related death and are thus the optimal candidates for an *active surveillance* strategy. This corresponds to the current EAU guidelines [9]. Active treatment is still recommended for all other patients since about 85% of even the small solid tumors ultimately turn out to be malignant [5]. Moreover, a *surveillance *strategy involves a small but significant risk of progressive local tumor growth that can potentially render nephron-sparing surgery difficult or impossible. In addition, even in patients with small tumors, *active surveillance *is associated with a 1-2% annual risk of systemic metastasis, and potentially curable patients may thus become incurable [10–12]. 

### 2.2. Renal Tumor Biopsy

The aim of renal tumor biopsy is to avoid unnecessary surgery in patients with renal findings. It is always indicated when the radiological diagnosis of RCC appears questionable (e.g., differential diagnosis including lymphoma, metastasis, or abscess) and should also precede thermal ablation. 

A number of studies published in 2011 dealt with the strengths and weaknesses of renal tumor biopsy [13–15]. They all showed a close correlation between biopsy and resection tissue: 91–93% for the histological subtype and 54–78% for the degree of tumor differentiation. Moreover, all studies revealed a very high positive predictive value (PPV) for renal tumor biopsy (95–100%) as well as relatively high specificity (69–100%) and an acceptable sensitivity (72–89%). However, the negative predictive value is still poor (NPV, 11.7 to 31%), which means that still a negative biopsy cannot reliably exclude a renal tumor and at least short-interval monitoring is required for patients with negative biopsies.

### 2.3. Radical versus Partial Nephrectomy

Woldrich et al. [16] from San Diego published a retrospective analysis of the NIS (*Nation wide Inpatient Sample*) data base in the USA, which included 615,500 patients with renal tumor interventions from 1998 to 2008. Radical nephrectomy was performed in 81.3% of these patients, partial nephrectomy in 13.7%, and local tumor ablation in 5.1% (cryotherapy or radiofrequency ablation). The proportion of nephron-sparing interventions increased over time but was still significantly lower than that of tumor nephrectomies, even among patients with chronic renal failure. 

At the 2011 Annual Meeting of the American Society of Clinical Oncology (ASCO), Kates et al. [17] from New York presented a SEER database analysis covering 4,216 RCC patients who underwent surgery between 1998 and 2007 for tumors <2 cm. Partial nephrectomies were performed in the majority of cases, but the proportion of radical nephrectomies was still high at 45%, even in this patient population with tumors <2 cm. However, the percentage of nephron-sparing interventions has increased significantly in recent years: from 27% in 1998 to 66% in 2007. Using multivariate analysis, Kates et al. [17] provided convincing evidence that radical nephrectomy *per se *significantly increases the mortality risk (HR: 2.24; 95% CI: 1.75–2.84). In the multivariate analysis, also the specific risk of cardiovascular mortality was significantly higher after radical than after partial nephrectomy (HR: 2.53; 95% CI: 1.51–4.23). 

Antonelli et al. [18], on the other hand, dealt with tumor-specific survival after partial and radical nephrectomy in patients with pT1 tumors. Within the framework of the SATURN project involving 16 Italian centers, they analyzed data of 3,320 pT1 patients surgically treated between 1995 and 2007. Their findings conclusively showed that tumor-specific survival was comparable after partial and radical nephrectomy. A synopsis of the data published by Kates et al. [17] and Antonelli et al. [18] thus clearly demonstrates longer overall survival after partial than after radical nephrectomy but comparable tumor-specific survival after both interventions. 

In order to completely exclude an oncological influence on the survival time after partial and radical nephrectomy, Childs et al. [19] performed a retrospective unicenter analysis of their patients who underwent surgery for an ultimately benign renal tumor between 1980 and 2008. The average follow-up was 8.3 years in 442 patients with a unilateral benign lesion. Using multivariate analysis, Childs et al. [19] were also able to show that even these patients had a 1.57 times higher risk of death after radical than after partial nephrectomy (*P* = 0.029). Thus radical nephrectomy was confirmed as an independent predictor of early death in this study as well.

To also elucidate the financial impact of the surgical procedure on the healthcare system, Chang et al. [20] used a cost analysis from the USA to demonstrate that performance of radical rather than partial nephrectomy in patients with pT1a RCC—regardless of their age—was associated with a significantly greater financial burden because of the higher incidence of chronic renal failure after surgery. Thus, radical nephrectomy resulted in 24%, 28%, and 31% higher healthcare costs than partial nephrectomy in 55-, 65-, and 75-year-old patients.

### 2.4. Access and Technique for Partial Nephrectomy

At the European Association of Urology (EAU) Congress in 2011, Sun et al. [21] presented a study that retrospectively illuminated NIS data from 1998 to 2007 with regard to complications associated with open versus laparoscopic renal tumor surgery. The study included 48,321 patients. In short, the authors were able to demonstrate that complication rates were comparable for open and laparoscopic partial nephrectomy but that laparoscopic radical nephrectomy was associated with significantly lower morbidity than open radical nephrectomy. 

A small retrospective monocenter study was presented by Hoff et al. [22] from Oslo on the occasion of the EAU Annual Meeting. Here 48 patients underwent either laparoscopic (*n* = 30) or robot-assisted (*n* = 18) partial nephrectomy between 2006 and 2010. Robot-assisted surgery was found to be superior with regard to the mean operating time (126 versus 163 min), the mean warm ischemia time (17 versus 24 min), and the mean blood loss (112 versus 305 ml). There is every indication that robot-assisted partial nephrectomy is technically easier and thus faster to perform than laparoscopic partial nephrectomy. In this small patient population, markedly fewer positive margins were seen after robot-assisted than after laparoscopic partial nephrectomy (11 versus 23%), though the overall rates seemed very high. 

To simplify the performance of partial nephrectomy, Schnoeller et al. [23] published a technique in which the capsular defect following open tumor enucleation of larger tumors is covered by modified porcine mucosa (Surgisis) over hemostatic material applied to the parenchyma. The aim was to evenly distribute pressure across the wound surface for optimal hemostasis without further nephron loss due to a puckered suture line. The authors concluded that this technique can achieve blood-tight and, if necessary, urine-tight closure of even large defects and helps to facilitate the performance of partial nephrectomies.

### 2.5. Local Ablation Procedure

Based on SEER database data from 1998 to 2007, Whitson et al. [24] compared the effectiveness of local ablation techniques with the current standard of partial nephrectomy for patients with pT1a RCC. They identified 8,087 patients who underwent partial nephrectomy (mean follow-up: 3.1 years) and 836 who were submitted to a local ablation procedure (mean follow-up, 1.9 years). Multivariate analysis identified the following independent negative predictors of tumor-specific survival: older age (HR: 1.9; 95% CI: 1.6–2.3), unmarried status (HR: 1.9; 95% CI: 1.3–2.9), and tumors with >1 cm growth rate (HR 1.3; 95% CI: 1.0–1.5). In addition, local ablation compared to partial nephrectomy was identified as an independent negative predictive factor for tumor-specific survival after the fourth *postintervention* year (HR: 4.8, 95% CI: 1.8–12.3). Whitson et al. [24] concluded that partial nephrectomy is the more effective type of treatment for patients with a life expectancy of ≥4 years.

Brausi et al. [25] published the first prospective follow-up study in patients with small renal tumors (<3 cm) who were treated with open partial nephrectomy, radiofrequency ablation (RFA), or an *active surveillance *strategy. All patients were older (>70 years) and had significant comorbidities (ASA 3/4). This prospective study with a homogeneous patient population also had a long follow-up in all subgroups, that is, of 60.1 months in patients with open partial nephrectomy (*n* = 27), 62.1 months in those with RFA (*n* = 24), and 56.3 months in those with *active surveillance* (*n* = 22). In the course of this mean follow-up period of about five years, 92.6% of the patients who were treated by nephron-sparing surgery were found to be completely tumor-free, whereas only 8.3% of those submitted to RFA no longer showed any signs of residual tumor. However, another 70.1% were classified as having *stable disease* in this group. On the other hand, significant local tumor progression was documented for three patients (12.5%) in the RFA and three (17%) in the *active surveillance* group. A limitation of the study lies in the fact that no tumor biopsies were performed prior to its initiation in either the RFA or the *active surveillance* group. Thus the inclusion of patients with benign renal tumors in these groups cannot be reliably ruled out. The authors concluded that partial nephrectomy should still remain the standard treatment for small renal tumors. A local ablation procedure or an *active surveillance* strategy is only seen as a genuine alternative for patients with a high perioperative risk or a short overall life expectancy due to significant comorbidities. 

A meta-analysis published in European Urology in 2011 by the study group of Tobias Klatte and Mesut Remzi from Vienna compared the results of partial nephrectomy (open or laparoscopic) with those of laparoscopic cryotherapy [26]. Laparoscopic cryotherapy was used for comparison with the standard procedure (partial nephrectomy) because it is still considered to be the most effective local ablation technique. This meta-analysis by Klatte et al. [26] included 1,295 patients who received cryotherapy and 5,347 who underwent partial nephrectomy. Cryotreated patients were significantly older (66.2 versus 60.6 years); they also had smaller tumors (2.4 versus 3.0 cm) and a markedly shorter follow-up (29.3 versus 57.3 months). Multivariate analysis of this large patient population showed that, even with a considerably shorter follow-up time, the cryotherapy group had a 5.24 times higher local postinterventional progression rate than the partial nephrectomy group (8.5% versus 1.9%). On the other hand, the cryotherapy group had a significantly lower complication rate than the partial nephrectomy group (17 versus 23%). 

The fact that even local ablation techniques are not risk-free, however, was demonstrated at the AUA Annual Congress by a group working with Psutka from Boston [27]. Here a retrospective analysis was performed to examine 274 patients with 311 renal tumors who underwent RFA between 1998 and 2008 (mean follow-up: 4.1 years). The peri-interventional complication rate was 19.3%; 14.2% of the complications were classified as minor ones and 5.1% as major ones according to the Clavien system. They included 7 ureteral strictures, 5 hemorrhages requiring transfusion, and 3 perirenal abscesses. One patient developed intestinal necrosis. Predictors of complications in this analysis were large tumor size and central tumor location. 

### 2.6. Prognostic/Predictive Factors for Localized RCC

Du et al. [28] from China assessed the prognostic value of preoperative plasma fibrinogen levels in patients with RCC. They performed a retrospective monocenter analysis in 286 patients who underwent radical nephrectomy for RCC between 2000 and 2003. Fibrinogen levels were found to correlate closely with tumor size. Moreover, multivariate analysis showed that a high fibrinogen level predicted metachronic metastases and poor tumor-specific survival. 

Johnson et al. [29] from Atlanta assessed the prognostic value of another acute-phase protein, C-reactive protein (CRP). They showed that high CRP values prior to radical nephrectomy could predict shorter survival for patients that had localized RCC at the time of surgery. However, it was not entirely understandable that the median survival times in this patient population with localized disease were only 43.4, 41.8, and 31.4 months for patients with a low, slightly increased and markedly increased CRP value. 

In another publication, Johnson et al. [30] investigated whether not only serum CRP but also intratumoral CRP expression is of prognostic relevance for survival after radical nephrectomy. This analysis showed that intratumoral CRP expression is also an independent prognostic marker for the survival of RCC patients. Patients who had tumors that expressed little or no CRP lived significantly longer (mean of 40.5 or 44.2 months) than those with high intratumoral CRP expression (31.6 months; HR: 11.9; 95% CI: 1.3–111.2). On the other hand, again, the short overall survival times were astonishing.

For the Italian SATURN project, Valotto et al. [31] analyzed the prognostic relevance of necrotic areas in renal tumor specimens. The median follow-up was 40 months in 2,719 patients with clear cell RCC. The histopathological specimens obtained from 21% of these patients contained necrotic areas. Multivariate analysis showed that the microscopically detectable tumor necrosis was significantly associated with poorer tumor-specific survival (HR: 1.7, *P* < 0.001). Accordingly, the 5-year tumor-specific survival was 89.4% without and 58.9% with tumor necrosis.

Margulis et al. [32] investigated whether the number of aberrantly expressed mTOR pathway components is of prognostic relevance for tumor-specific survival of patients with RCC. Specimens obtained from 258 clear cell RCC patients between 1997 and 2008 were retrospectively examined. A prognostic score was developed based on the number of aberrant mTOR pathway components (mTOR, Raptor, p-4E-BP1, P13 kinase, and PTEN). Detection of less than three altered components was classified as a favorable biomarker, while three or more altered components indicated an unfavorable prognosis in univariate analysis. Multivariate analysis also revealed that several alterations in the mTOR pathway tended to be associated with reduced tumor-free survival (HR: 2.9; *P* = 0.06).

The following are take-home messages regarding localized RCC.

- The risk of malignancy and metastatic spread as well as the aggressive potential of a localized solid renal tumor increases continuously with size.
- The active surveillance strategy as well as local ablation techniques can currently be recommended only in a selected patient population (older patients with significant comorbidities).
- Today, renal tumor biopsy is relatively harmless and has a high positive predictive value, though its negative predictive value is still low.
- If oncologically acceptable and technically feasible, nephron-sparing surgery should always be performed, regardless of tumor size. The access and surgical technique for partial nephrectomy should be geared mainly to the surgeon's experience.
- Possible prognostic parameters for localized RCC published in 2011 were among others: alterations in the mTOR signal transduction pathway, microscopically visible intratumoral necrotic areas, and high preoperative CRP and fibrinogen values.

## 3. Advanced RCC

### 3.1. (Partial) Nephrectomy in Patients with Metastatic Disease

At the EAU Congress in Vienna, Hussain et al. [33] from London presented a retrospective analysis comparing perioperative morbidity of 28 nonmetastatic with that of 22 metastatic RCC patients after preoperative sunitinib therapy. The authors concluded that surgery is technically more complex and difficult after upfront sunitinib therapy: the sunitinib-pretreated group had a significantly higher median blood loss (775 ml versus 320 ml), a longer operating time (195 min versus 128 min), and a higher number of intraoperative complications (5 versus 2). On the other hand, wound-healing complications were not more common in the sunitinib-pretreated group. 

The oncological value of nephrectomy in patients with metastatic spread is currently being evaluated in two large prospective studies (the CARMENA Study and the SURTIME Study). To make a definitive statement here, we will surely have to wait for an analysis of these studies. Already in 2011, however, several study groups examined the impact of cytoreductive nephrectomy on the overall survival of patients treated with *targeted drugs*. Crepel et al. [34] published a retrospective multicenter analysis in which 18 centers enrolled 351 patients who were treated with different antiangiogenic agents. Here the patients who underwent nephrectomy in addition to systemic antiangiogenic therapy lived significantly longer than those who were not nephrectomized (median: 38.1 months versus 16.4 months; *P* < 0.001). This survival advantage gained by nephrectomy was particularly marked in patients with an ECOG performance status of 0-1 (43.3 versus 16.7 months, *P* = 0.03). However, the difference in overall survival between patients with and without nephrectomy was no longer statistically significant for those with an ECOG performance status of 2-3 (12.6 versus 8.0 months; *P* = 0.8). Even using the MSKCC risk group stratification, mainly MSKCC good- or intermediate-risk patients derived benefit from nephrectomy in this large retrospective analysis (42.4 months with and 16.8 months without nephrectomy; *P* = 0.02). Here too, overall survival did not differ among poor-risk patients, regardless of whether or not they were nephrectomized (5.2 months in either case).

Nozawa et al. [35] performed a retrospective unicenter study comparing the response of *in situ* primary tumors and corresponding metastases to targeted systemic therapy. Of the 17 patients included, 35% received first-line treatment with sunitinib, 35% with sorafenib, and 6% each with pazopanib and everolimus. The median maximum decrease in tumor size was 6% (−48% to 13%) for the primary RCC and 10% (−100% to 76%) for the metastases. In 29% of the patients, the primary tumor and metastases even had the opposite response to systemic tumor therapy. The authors concluded that (a) the response of the primary tumor and metastases very often differ markedly, (b) the metastases usually respond to systemic therapy somewhat better than the primary tumor itself, and thus (c) the response of the metastases cannot be used to draw conclusions about the response of the primary tumor.

### 3.2. Systemic Treatment Options

At the last ASCO Annual Meeting, Melichar et al. [36] presented an open-label, single-arm, prospective multinational phase II study (BEVLiN study) in which 147 nephrectomized MSKCC good- and intermediate-risk patients with clear cell RCC received bevacizumab combined with low-dose IFN*α* (3 MIU). This patient population was compared with an AVOREN subgroup (*n* = 272) in which patients were treated with a higher IFN*α* dose (9 MIU). The median follow-up was 15.3 months in this study. Median progression-free survival was longer for patients in the BEVLiN study than for those in the control arm or the AVOREN trial (14.8 versus 10.5 months). Overall survival also tended to be longer for patients in the BEVLiN study (HR: 0.74; 95% CI: 0.51–1.08). These data were surprising in view of the fact that the response rate was markedly lower in the BEVLiN than in the AVOREN study (24.3% versus 35.9%).

Busch et al. [37] performed a bicentric study to evaluate the response of patients to an mTOR inhibitor after failure of treatment with one or more tyrosine kinase inhibitors. In this analysis, 32 patients were treated with everolimus and 29 with temsirolimus. In this retrospective nonrandomized analysis, both substances—everolimus and temsirolimus—showed comparable effectiveness with median progression-free periods of 5.9 and 5.1 months as well as median overall survival times of 18.7 and 18.0 months. Disease stabilization was achieved in 53% of the patients with everolimus and in 52% with temsirolimus. The rate of grade 3-4 side effects did not differ significantly either (44 and 34%). 

Presentation of the first results of the Axis trial was one of the urooncology congress highlights of the ASCO Annual Meeting this year in Chicago [38]. This international prospective randomized phase III study tested the tyrosine kinase inhibitor (TKI) axitinib against sorafenib for effectiveness and toxicity in randomized patients who progressed after first-line therapy. Previous phase II studies had confirmed the effectiveness of axitinib—another TKI that targets mainly VEGFR, PDGFR, and c-KIT—after cytokine and sorafenib failure. A total of 723 patients were randomized 1 : 1 to receive axitinib or sorafenib. They were treated with sorafenib 2 × 400 mg/day or axitinib 2 × 5 mg/day with the option of increasing the latter to 2 × 10 mg/day if well tolerated. The study included patients with metastatic clear cell RCC who were in good general condition (ECOG performance status 0-1) and had received previous therapy: sunitinib (54%), cytokines (35%), bevacizumab (8%), and temsirolimus (3%) in both arms. The MSKCC risk scores were also evenly distributed: 40% favorable, 54% intermediate, and 2% poor. 

For the total group, progression-free survival was 6.7 months in the axitinib arm and 4.7 months in the sorafenib arm (HR: 0.67, *P* < 0.001). The partial remission rate was also significantly higher in the axitinib arm (19.4% versus 9.4%). There were no differences in the number of stable diseases (49.9% versus 54.4%) or patients with primary progressive disease (21.6% versus 21.0%). The mean dose intensity was 98.6% and 91.7%. However, it should be noted here that, in the course of the study, the dosage was increased at least temporarily in 36.8% of the axitinib patients. The safety profile was comparable for both therapies. The following seemed to occur more frequently in the axitinib arm: grade 3/4 gastrointestinal side effects (diarrhea 11 versus 7%, nausea and vomiting 7 versus 2%), arterial hypertension (16 versus 11%), and fatigue (11 versus 5%). On the other hand, sorafenib was more often associated with more severe hand-foot syndromes (16 versus 5%) and erythema (4 versus <1%).

In summary, the first results of the Axis trial clearly confirmed the effectiveness of axitinib (and sorafenib) in the (second-line) therapy of metastatic clear cell RCC. It seems to be a safe drug with toxicity comparable to that of other TKIs. It achieved significantly better results than sorafenib in terms of objective remissions and progression-free survival. The latter improvement was most marked in the subgroup of cytokine-pretreated patients (approx. 5 months), while a smaller difference was seen after failure of sunitinib (approx. 1.5 months). 

The Axis trial yielded another very important result: it was the first prospective phase III study to demonstrate that the TKI-TKI-sequence is a sensible and effective treatment option for patients with advanced renal cell carcinoma. Several limitations should be noted: (a) the study was not blinded, (b) local and central radiology yielded in part dramatically conflicting results, and (c) only axitinib could be dose-escalated, although a number of publications have already confirmed that a dose escalation of other TKIs can also lead to a higher response rate and longer progression-free survival. 

Reeves et al. [39] presented a small nonrandomized multicenter phase II study in which 46 patients received pazopanib for second-line treatment of metastatic RCC after progression or intolerance under first-line treatment with sunitinib (*n* = 34) or bevacizumab (*n* = 12). *Second-line* pazopanib led to partial remission in 22% of all patients and to disease stabilization in another 48%. The median progression-free survival in the total patient population was 9 months (5.3 to 11.9 months). The 12-month survival probability was 64%. The most common side effects of pazopanib for second-line treatment corresponded to those known to occur during *first*-*line *treatment: grade 3/4 toxicity including fatigue (15%), arterial hypertension (11%), and proteinuria (11%). The authors concluded that *second-line *use of pazopanib after intolerance or progression of *first-line* antiangiogenetic treatment appears to be effective and well tolerated and that, in the future, pazopanib will also play a role in the sequence of VEGF-targeted therapies.

Kapadia et al. [40] conducted a meta-analysis of 8 prospective studies to assess the hepatotoxicity of pazopanib monotherapy (800 mg/d). The study population comprised 1,155 patients with various solid tumors, including RCC. An ALT increase was seen in 41.7% of the patients, and 8.2% even had a high-grade increase. Interestingly, the incidence of pazopanib-related high-grade ALT increases differed significantly between patients with RCC and those with other solid tumors (10.9 versus 5.7%; *P* = 0.01). AST increases were seen in 39.3% of the patients and high-grade increases in 6.4%. Here no significant difference was found between patients with RCC and those with other malignant diseases (7.4% versus 4.8%, *P* = 0.22). The authors concluded that pazopanib therapy is associated with a substantial risk of significant hepatotoxicity and thus requires close monitoring. It remains unclear why the incidence of pazopanib-related hepatotoxicity seems to be higher in patients with RCC than in those with other tumors. 

The following are take-home messages regarding advanced RCC.

- Neoadjuvant or “downsizing preoperative” targeted therapy does not yet play a clearly defined role in the management of advanced/metastatic RCC.
- IFN used in combination with bevacizumab for first-line treatment of MSKCC good- and intermediate-prognosis patients with clear cell RCC can (and should) be given at a lower dose.
- Sequence therapy with tyrosine kinase inhibitors is an effective alternative to the TKI/mTOR inhibitor sequence.
- Pazopanib also appears to be another effective TKI also for second-line treatment.
- A positive phase III study confirmed that axitinib has the potential for a standard second-line treatment, but it still lacks approval.
- No prognostic/predictive markers applicable in routine clinical practice are in the offing for patients with RCC or for a specific treatment.
